# Sequence divergence of *Mus spretus *and *Mus musculus *across a skin cancer susceptibility locus

**DOI:** 10.1186/1471-2164-9-626

**Published:** 2008-12-23

**Authors:** Kimberly L Mahler, Jessica L Fleming, Amy M Dworkin, Nicholas Gladman, Hee-Yeon Cho, Jian-Hua Mao, Allan Balmain, Amanda Ewart Toland

**Affiliations:** 1 Division of Human Cancer Genetics, Department of Molecular Virology, Immunology and Medical Genetics, OSU Comprehensive Cancer Center, The Ohio State University, OH, USA; 2 Molecular, Cellular and Developmental Biology Graduate Program, The Ohio State University, OH, USA; 3 Integrated Biomedical Science Graduate Program, The Ohio State University, OH, USA; 4 Cancer Research Institute, University of California, San Francisco, San Francisco, CA, USA; 5 Division of Human Genetics, Department of Internal Medicine, The Ohio State University, Columbus, OH, USA

## Abstract

**Background:**

*Mus spretus *diverged from *Mus musculus *over one million years ago. These mice are genetically and phenotypically divergent. Despite the value of utilizing *M. musculus *and *M. spretus *for quantitative trait locus (QTL) mapping, relatively little genomic information on *M. spretus *exists, and most of the available sequence and polymorphic data is for one strain of *M. spretus*, Spret/Ei. In previous work, we mapped fifteen loci for skin cancer susceptibility using four different *M. spretus *by *M. musculus *F1 backcrosses. One locus, *skin tumor susceptibility 5 *(*Skts5*) on chromosome 12, shows strong linkage in one cross.

**Results:**

To identify potential candidate genes for *Skts5*, we sequenced 65 named and unnamed genes and coding elements mapping to the peak linkage area in outbred *spretus*, Spret/EiJ, FVB/NJ, and NIH/Ola. We identified polymorphisms in 62 of 65 genes including 122 amino acid substitutions. To look for polymorphisms consistent with the linkage data, we sequenced exons with amino acid polymorphisms in two additional *M. spretus *strains and one additional *M. musculus *strain generating 40.1 kb of sequence data. Eight candidate variants were identified that fit with the linkage data. To determine the degree of variation across *M. spretus*, we conducted phylogenetic analyses. The relatedness of the *M. spretus *strains at this locus is consistent with the proximity of region of ascertainment of the ancestral mice.

**Conclusion:**

Our analyses suggest that, if *Skts5 *on chromosome 12 is representative of other regions in the genome, then published genomic data for Spret/EiJ are likely to be of high utility for genomic studies in other *M. spretus *strains.

## Background

*Mus spretus *mice are derived from wild mice collected in Southern France, Spain and Northern Africa [1,2]. *M. spretus *diverged from *Mus musculus *one to three million years ago[3,4]. Recent data suggest that these mice are reported to have an average of one sequence variant every 50 bp [5]. Strains of *M. spretus *are frequently utilized in combination with *M. musculus *strains in quantitative trait loci (QTL) studies due to the high degree of sequence and phenotypic diversity between the strains[6,7]. Indeed, *M. spretus *mice have been valuable for the identification of loci contributing to differences in immune response and inflammation [8-10]. *M. spretus *strains are also cancer resistant in comparison to several *M. musculus *strains and have been utilized for mapping of cancer susceptibility loci [11-16].

Unlike some of the other commonly used strains of mice for QTL mapping, none of the *M. spretus *strains have been fully sequenced. Few reports of comparisons of sequence diversity within *M. spretus *strains exist, and most have been generally limited to one gene or small genomic region [17-19]. The majority of the sequence, microsatellite, and single nucleotide polymorphism (SNP) data available for *M. spretus *mice are from Spret/Ei; for researchers using non-Spret/Ei strains of *M. spretus*, such as SEG/PAS mice, there is limited strain-specific information available in public databases.

In previous studies, we observed a large difference between *M. musculus *and *M. spretus *strains in susceptibility to chemically-induced skin cancer. *M. spretus *mice chemically treated with 7, 12-dimethylbenz [a]anthracene (DMBA) and 12-O-tetradecanoylphorbol-13-acetate (TPA) develop no skin papillomas after 20 weeks of treatment but *M. musculus *strains, such as NIH/Ola and FVB/N, develop an average of 20 or more papillomas [11]., To identify loci for skin cancer susceptibility we created four F1 backcrosses between *M. spretus *(Spret/EiJ, STF/PAS, outbred *spretus*) and *M. musculus *(FVB/NT, NIH/Ola) mice. Fifteen regions of linkage were identified [11-13,20,21]. One locus, *skin tumor susceptibility 5 *(*Skts5*), a region on chromosome 12 with peak linkage at 17 cM showed strong evidence of linkage in one F1 backcross NIH/Ola × F1(NIH/Ola × outbred *spretus*).

As one means of identifying potential skin cancer susceptibility candidate genes at *Skts5*, we sequenced all predicted genes in four of the strains used for linkage NIH/Ola, FVB/NJ, outbred *spretus*, and Spret/EiJ. Exons that contained amino acid alterations between the strains were sequenced in two additional strains of *M. Spretus *(SEG/PAS and STF/PAS) as well as FVB/NT (FVB/N mice, Taconics). Here, we describe the results from over 140 kb of sequence data from *Skts5*, 40.1 kb of which was sequenced in seven strains, and a phylogenetic comparison of the relatedness of four *M. spretus *strains.

## Results

### Confirmation of *Skts5 *as a skin tumor susceptibility locus

**Figure 1:**
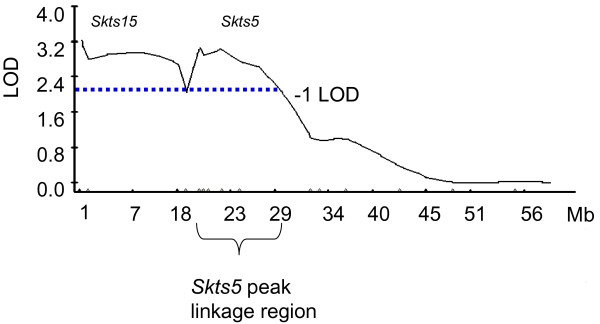
**Linkage data at Skts5**. Eighteen markers mapping to chromosome 12 were genotyped in 351 NIH/Ola × (NIH/Ola × *spretus *outbred) backcross mice. Analysis using WinQTLcart shows two peak linkages. *Skts15 *maps to proximal chromosome 12 and *Skts5 *maps to chromosome 12 with a peak linkage at ABI marker E_29.924 (35 Mb, 17 cM). The one negative lod confidence interval is marked by a dotted line.

*Skin tumor susceptibility 5 *(*Skts5*) was originally mapped as a skin tumor susceptibility locus to mouse chromosome 12 in a genome wide linkage analysis for skin cancer in an F1 backcross between NIH/Ola and outbred *spretus *mice [11,12]. We performed additional crosses between skin tumor resistant mice (Spret/EiJ, STF/PAS) and skin tumor susceptible mice (NIH/Ola, FVB/NT) to further test this locus for linkage [13,20,21]. We observed no evidence for linkage at *Skts5 *in the interspecific backcross NIH/Ola × [STF/PAS × NIH/Ola] or in the Spret/EiJ by FVB/NT F1 interspecific backcross. The linkage analysis for the Spret/EiJ by NIH/Ola F1 interspecific backcross (approximately 80 mice) was inconclusive for linkage at *Skts5*. To refine the linkage region in the NIH/Ola × outbred *spretus *backcross, we genotyped additional markers on chromosome 12. Linkage analyses showed a peak LOD score of 3.2 at approximately 17 cM (35 Mb; Figure 1).

Outbred *spretus *mice are not bred to homogeneity and segregate different polymorphisms and haplotypes across the genome. However, there was no evidence from the original linkage data generated from nine different outbred *spretus *fathers using 18 polymorphic markers for heterogeneity at *Skts5*. To further rule out the possibility of heterogeneity within these mice at *Skts5*, we genotyped seven additional microsatellite markers mapping to the peak linkage region of *Skts5 *in DNA from six non-littermate outbred *spretus *male mice. We observed no evidence of genetic heterogeneity (data not shown). From these results we felt reasonably confident that there was no genetic heterogeneity within our outbred *spretus *mouse colony at this locus.

### Identification of variants between *M. musculus *and *M. spretus*

In order to identify polymorphic coding variants that fit the linkage data for *Skts5*, we first identified all known coding and non-coding genes at the peak region for linkage on chromosome 12 (between 31.7 and 47.9 Mb, Ensembl build 50) using genetic maps from the UCSC Genome Browser, Ensembl and Entrez Gene databases. Maps were updated and throughout our project to include newly mapped genes. At the conclusion of this study, 65 coding elements including ten hypothetical genes/coding elements, nine snoRNAs and one microRNA were identified as mapping within *Skts5 *(see Additional file 1). We designed PCR primers flanking all exons for the genes or coding elements mapping to *Skts5*. When multiple splice forms of a gene were predicted, we sequenced exons representing all splice variants.

To carry out a screen for coding differences between the resistant strain of mice (outbred *spretus*) and the susceptible strain (NIH/Ola) that could be candidate variants for *Skts5*, we initially sequenced DNA from NIH/Ola, FVB/NJ, Spret/EiJ, and one outbred *spretus *animal. Exons containing amino acid variants were then sequenced in FVB/NT, SEG/PAS and STF/PAS. Variants shared between outbred *spretus *and STF/PAS were eliminated as candidates since STF/PAS does not show evidence for linkage at *Skts5 *in the NIH/Ola backcross. PCR product was obtained from both *M. spretus *and *M. musculus *DNA for all exons which rules out the possibility of large deletions in one strain of mice at this locus. Our analysis did not rule out the possibility of causal variants in regulatory regions or duplication or inversion copy number polymorphisms at this locus.

Analysis of sequence alignments of forward and reverse reads of 140,448 bp from genes and coding elements mapping to *Skts5 *led to the identification of 1,123 polymorphisms between *M. musculus *and *M. spretus *strains (Tables 1, 2 and Additional file 2). Of these, 122 resulted in amino acid substitutions, 121 occurred between *M. musculus *and *M. spretus *strains and one occurred between NIH/Ola and FVB/NJ. There were six sequence differences identified between FVB/NJ and NIH/Ola, but no differences between FVB/NT and NIH/Ola.

**Table 1:** Amino acid variants within *M. spretus *strains

Gene	Variant	EiJ	Outbred	SEG	STF	NIH/FVB
*Lamb1-1*	Gly1334Asn	Asn	Asn	Gly	Asn	Gly

*Cog5*	Asp538Glu	Glu	Glu	Asp	Glu	Asp
	
	Asp742Glu	Glu	Glu	Glu	Asp	Asp

*1110049B09rik*	Pro691Ala	Ala	Pro	Pro	Ala	Pro

*F730043M19Rik*	Ser115Leu	Leu	Leu	Ser	Leu	Ser
	
	Asn133Ile	Ile	Asn	Asn	Asn	Asn

*Twistnb*	Asn322Asp	Asp	Asn	Asp	Asn	Asn

*Hdac9*	Ile265Met	Met	Met	Ile	Ile	Ile
	
	Gly749Ser	Ser	Gly	Gly	Gly	Gly
	
	Ala994Val	Val	Ala	Val	Ala	Ala

*Ahr*	Asn533Ser	Ser	Ser	Asn	Asn	Asn
	
	Asn544Asp	Asp	Asp	Asn	Asn	Asn

*EG629820*	Gly47Ser	Ser	Gly	Gly	Ser	Gly
	
	Gly70Ser	Ser	Gly	Gly	Ser	Gly
	
	Asn72Tyr	Asn	Tyr	Tyr	Asn	Asn

*Zfp277*	Gln21Phe	Phe	Phe	Val	Val	Gln
	
	Val434His	His	His	Gln	His	Val

*Lrrn3*	Ala69Gln	Gln	Gln	Gln	Ala	Ala
	
	Leu670Val	Val	Val	Val	Leu	Leu
	
	Ala671Val	Val	Ala	Ala	Ala	Ala

*DnaJB9*	Phe10Leu	Leu	Phe	Phe	Phe	Phe

*NrCam*	Gln152Arg	Arg	Gln	Gln	Arg	Gln

**Table 2:** Potentially functionally significant amino acid variants

Gene	Amino Acid	MM	MS	HS	RN	GG	BT	CF	XT	MD	TR	SIFT
*Slc26a3*	His38Asn	His	Asn	His	His	His	His	His	His	His	His	0.13

*Dus4L*	Cys104Tyr	Cys	Tyr	Cys	Cys	Asn	Cys	Cys	Ser	Cys	Ala	0.18

*Cog5*	Ser769Ala	Ser	Ala	Ser	Ser	Ser	Ser	Ser	NH	Ser	Ser	0.09

*1110049B09Rik*	Pro691Ala	Pro	Pro/Ala	Pro	Lys	Pro	Pro	Pro	Pro	Pro	NH	0.11

*F730043M19Rik*	Asn133Ile	Asn	Ile/Asn	NH	NH	NH	NH	NH	NH	NH	NH	**0.00**

*4930504H06Rik*	Met64Val	Met	Val	Met	Met	NH	Met	Met	NH	Met	Phe	0.13

*Ahr*	Leu348Phe	Leu	Phe	Leu	Leu	Leu	Leu	Leu	Leu	Leu	Leu	**0.01**
	
	Ser790Gly	Ser	Gly	Asn	Ser	Asp	Asn	Asn	Asn	His	NH	0.15

*Ankmy2*	Ile369Thr	Ile	Thr	Ile	Thr	Leu	Leu	Leu	Glu	Leu	NH	**0.03**

*Sostdc1*	Cys15Ser	Cys	Ser	Cys	Cys	Cys	Cys	Cys	Cys	Cys	Cys	**0.00**

*Ifrd1*	Leu449Phe	Leu	Phe	Phe	NH	Phe	Phe	Phe	Leu	Phe	Phe	**0.00**

*EG629820*	Gly47Ser	Ser	Ser/Gly	NH	NH	NH	NH	NH	NH	NH	NH	**0.00**
	
	Gly70Ser	Gly	Ser/Gly	NH	NH	NH	NH	NH	NH	NH	NH	**0.00**
	
	Asn72Tyr	Asn	Tyr/Asn	NH	NH	NH	NH	NH	NH	NH	NH	**0.00**

*Zfp277*	Val21Phe	Val	Val/Phe	Ser	NH	NH	Asp	Asp	NH	NH	NH	0.08
	
	Gln43His	Gln	Gln/His	Gln	NH	Gln	Gln	Gln	Gln	Gln	Gln	0.15

*Immp2L*	Ala8Val	Ala	Val	Val	Val	Gly	Val	Val	NH	Gly	Gly	0.15

*Lrrn3*	Ala69Gln	Ala	Ala/Gln	Ala	Ala	Ala	Ala	Ala	NH	Ala	Leu	0.08
	
	Ala671Val	Ala	Ala/Val	Ala	Ala	Ser	Ala	Thr	NH	Ala	NH	0.15

*Stxbp6*	Ile23Val	Ile	Val	Val	Val	Val	NH	Val	Val	Val	Val	0.12

Bold indicates significant SIFT scores. No known homology (NH), *Mus musculus *(MM), *Mus spretus *(MS), *Homo sapiens *(HS), *Rattus norvegicus *(RN), *Gallus gallus *(GG), *Bos Taurus *(BT), *Canis familiaris *(CF), *Xenopus tropicalis *(XP), *Monodelphis domestica *(MD), and *Takifugu rubripes *(TR). Sift scores in bold are predicted to be non-tolerated changes.

### Characterization of nucleotide substitutions at *Skts5*

**Figure 2:**
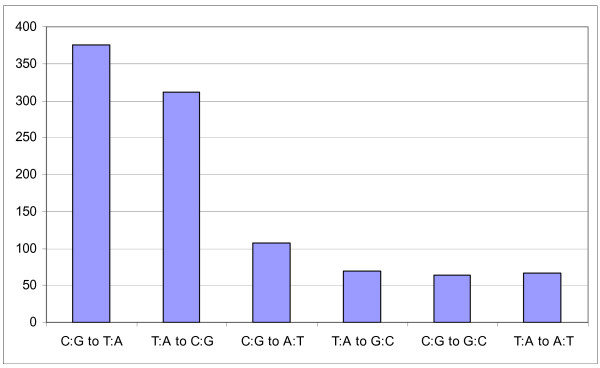
**Type and number of nucleotide substitutions**. The number of each type of single nucleotide polymorphism identified at *Skts5 *between NIH/Ola and Spret/EiJ is listed. The transition to transversion rate is roughly 2-fold.

To further characterize the nucleotide variants, we analyzed polymorphisms on a gene by gene basis. The number of polymorphisms identified per gene ranged from no changes to 65. The rate of substitution per nucleotide ranged from 3/3593 (0.08%) in *Nova1 *to 8/133 (6%) in *SNORNA17 *(Ensembl *ENSMUSG00000077294*). Because synonomous changes are less likely to affect protein function, these were not evaluated further in the other strains of mice used in the linkage crosses [22-24]. A catalogue of the types and locations of polymorphisms (Table 3: see Additional file 2) at Skts5 include the following changes: nonsense (1), missense (122), small insertion/deletions (14), 5'UTR (89), 3'UTR (404) and potential splice-site alterations (23). To determine if the type of substitutions identified at *Skts5 *could shed light on the evolutionary forces between *M. spretus *and *M. musculus*, we looked at the number and percentages of nucleotide changes and the type of mutations (transitions versus transversions) in this data set (see Additional file 2). The transition to transversion ratio was approximately 2.0 (Figure 2). Given that C to T (G to A) transitions are a common mutation type due to the CpG methylation deamination process; these data are consistent with other large sequence comparisons[25,26].

**Table 3:** Characteristics of nucleotide substitutions at *Skts5*

	5'UTR	Neutral	AA	3'UTR	Potential splice sites	SnoRNA	Total	Frequency of substitution/nt
C:G to T:A	27	203	41	140	13	10	434	0.00309

T:A to C:G	30	142	40	123	6	14	355	0.00253

C:G to A:T	7	26	19	52	0	6	110	0.00078

T:A to G:C	9	26	7	29	0	2	73	0.00052

C:G to G:C	8	19	18	25	1	2	73	0.00052

T:A to A:T	9	9	7	35	3	1	64	0.00046

total	90	425	132	404	23	35	1109	0.0079

Frequency of substitution/nt	0.00064	0.00303	0.00094	0.00288	0.00016	0.00025	0.0079	

AA = amino acid substitution, UTR = untranslated region, nt = nucleotide

### Identification of variants within *M. spretus*

We rationalized that in order for a bi-allelic polymorphism to be considered a candidate for *Skts5 *that the cancer resistant strain (outbred *spretus*) would have one allele and the susceptible strain (NIH/Ola) would have the other. In addition, closely related strain STF/PAS that does not show evidence for linkage at *Skts5 *should not share alleles with outbred *spretus*. Of the 121 amino acid substitutions identified between *M. musculus *and *M. spretus*, three variants were present in Spret/EiJ but not the other *M. spretus *strains (Table 1). To rule out a sequencing or PCR artifact for those variants, new PCR product was generated and sequenced. All variants were confirmed as being unique to Spret/EiJ. Of the other 118 amino acid variants which were polymorphic between NIH/Ola and *M. spretus*, 99 were shared in all *M. spretus *strains analyzed and 19 were polymorphic between different *M. spretus *strains (Table 1). Eight variants fit our criteria for candidacy based on the linkage data (Table 4).

**Table 4:** Candidate variants for *Skts5*

Gene	Variant	NIH/Ola	Outbred *spretus*	STF/PAS	SIFT
*Cog5*	Asp742Glu	Asp	Glu	Asp	0.92

*Hdac9*	Ile265Met	Ile	Met	Ile	0.33

*Ahr*	Asn533Ser	Asn	Ser	Asn	1.00

	Asn544Asp	Asn	Asp	Asn	0.66

*EG629820*	Asn72Tyr	Asn	Tyr	Asn	0.00

*Zfp277*	Gln21Phe/Val	Gln	Phe	Val	0.08

*Lrrn3*	Ala69Gln	Ala	Gln	Ala	0.08

	Leu670Val	Leu	Val	Leu	0.23

### Conservation of amino acid alterations

Just as non-synonomous changes have a greater likelihood of affecting protein function than synonomous changes, non-synonomous changes that occur in a evolutionarily conserved residue are more likely to be functionally significant [27,28]. To assess amino acid polymorphisms for potential functional significance, proteins were compared using Ensembl ortholog prediction alignments between *M. musculus *and *Homo sapiens*, *Pan troglyodytes*, *Gallus gallus*, *Rattus norvegicus*, *Canis familiaris*, *Bos Taurus*, *Xenopus tropicalis, Takifugu rubripes*, and *Monodelphis domestica *when protein homologies existed . If only one amino acid in the orthologous protein position was present in at least eight of the nine non-*musuclus *species examined, we considered that position to be strongly conserved. Twenty-four amino acids showed strong conservation by this criterion (see Additional file 3).

In addition to sequence alignments, we assessed amino acid polymorphisms for potential functionality through a sequence homology-based tool Sorting Intolerant From Tolerant (SIFT)[28]. Using SIFT, the majority of amino acid polymorphisms were scored as tolerant substitutions. Surprisingly, a few amino acids that appear to be highly conserved across species, such as *Pik3cg *Ala405Thr, were scored as well-tolerated substitutions using SIFT. Eight variants were identified that were scored as intolerant substitutions by SIFT (scores 0.05 or less). These variants include *Ahr *Phe348Leu, *Ankmy2 *Ile369Thr, *Sostdc1 *Cys15Ser, *Ifrd1 *Leu449Phe, *EG629820 *Gly47Ser, Gly70Ser and Asn72Tyr and *F730043M19Rik *Asn133Ile. Of these, we identified three (*Ahr *Phe348Leu, *Sostdc1 *Cys15Ser and *Ifrd1 *Leu449Phe) in our initial ortholog comparison studies (see Additional file 3). Seven additional variants in other genes had SIFT scores of less than 0.15 and may have mild functional effects (Table 2).

Of the 22 amino acid alterations that were polymorphic within *M. spretus *strains, four showed amino acid substitutions that were considered to be intolerant using SIFT (Table 5). These changes all occurred in genes for which there is limited homology data for other species which may lead to over prediction of intolerance by SIFT. Five other substitutions resulted in SIFT scores of 0.15 or lower including *1110049B09Rik *Pro691Ala and *Lrrn3 *Ala69Gln. Polymorphisms in highly conserved amino acids may result in functional differences in these proteins and could account for phenotypic differences between *M. spretus *strains.

**Table 5:** Evolutionary conservation of variants seen between *M. Spretus *strains

Gene	Variant	HS	RN	GG	BT	CF	XT	MD	SIFT
*Lamb1-1*	Gly1334Asn	Asp	Asp	NH	NH	Asp	NH	NH	0.47

*Cog5*	Asp538Glu	Asp	Asp	Asp	Asp	Asp	NH	Glu	1

	Asp742Glu	Ser	NH	Asn	Ser	Ser	NH	Arg	0.92

*1110049B09rik*	Pro691Ala	Pro	Lys	Pro	Pro	Pro	Pro	Pro	0.11

*F730043M19Rik*	Ser115Leu	NH	NH	NH	NH	NH	NH	NH	0.21

	Asn133Ile	NH	NH	NH	NH	NH	NH	NH	**0.00**

*Twistnb*	Asn322Asp	Glu	Asn	Glu	Glu	Glu	Asn	Leu	0.39

*Hdac9*	Ile265Met	Ile	Ile	Leu	Thr	Ile	Leu	Leu	0.33

	Gly749Ser	Gly	Gly	Gly	Gly	NH	NH	Ala	1.00

	Ala994Val	Val	Ala	Ile	Val	NH	NH	Ile	1.00

*Ahr*	Asn533Ser	Ser	Ser	Ser	Asn	Ser	Asn	Leu	1.00

	Asn544Asp	Asp	Asp	Asn	Phe	Asp	Asp	Gly	0.66

*EG629820*	Gly47Ser	NH	NH	NH	NH	NH	NH	NH	**0.00**

	Gly70Ser	NH	NH	NH	NH	NH	NH	NH	**0.00**

	Asn72Tyr	NH	NH	NH	NH	NH	NH	NH	**0.00**

*Zfp277*	Gln21Phe	Gln	NH	Gln	Gln	Gln	Gln	NH	0.15

	Val434His	Ser	NH	NH	Asn	Arg	NH	Asp	0.08

*Lrrn3*	Ala69Gln	Ala	Ala	Ala	Ala	Ala	NH	Gln	0.08

	Leu670Val	Phe	Leu	Ile	Phe	Phe	NH	Ile	0.23

	Ala671Val	Ala	Ala	Ser	Ala	Thr	NH	Phe	0.15

*DnaJB9*	Phe10Leu	Phe	Phe	Phe	Phe	Phe	Phe	Ala	1.00

*NrCam*	Gln152Arg	Gln	Arg	Arg	Arg	Arg	Gln	Phe	0.49

Bold indicates significant SIFT scores. No known homology (NH), No sift score generated (NSS) *M. musculus (MM*) *Homo sapiens (HS), Rattus norvegicus (RN)*, *Gallus gallus (GG)*, *Bos Taurus (BT)*, *Canis familiaris (CF)*, *Xenopus tropicalis (XP)*, and *Monodelphis domestica (MD)*.

### Polymorphisms at splice sites

Variants that fall at highly conserved splice acceptor or splice donor sites may also affect protein function due to alterations in splicing. Twenty-four variants mapped within 3 bp of a splice site. To test whether these variants affected splicing, we isolated tail RNA from NIH/Ola, outbred *spretus *and Spret/EiJ and performed RT-PCR using primers from flanking exons. Identically sized bands of roughly the same intensity were observed in all of the strains for nineteen of the primer sets tested indicating that these variants are unlikely to affect splicing (data not shown). Five variants were not tested for effects on splice site alterations because the variations mapped to the first or last exon of the gene.

### Comparison of sequence divergence within *M. Spretus *strains

**Figure 3:**
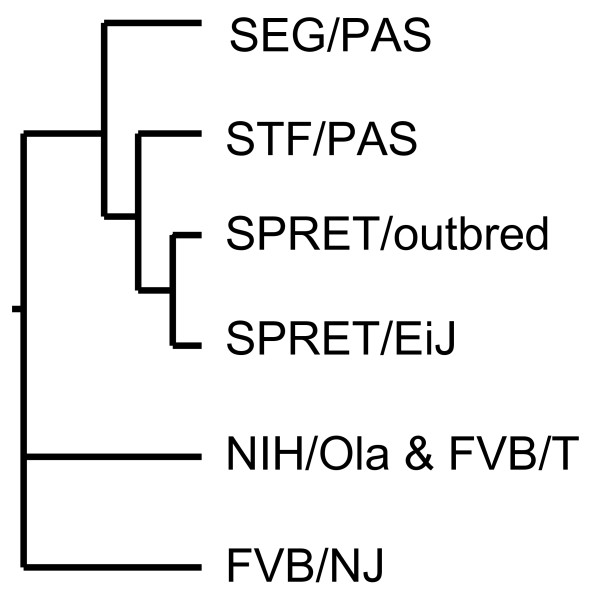
**Phylogenetic tree from sequence data at Skts5**. Phylogenetic tree generated by parsimony analysis of four *M. Spretus *and two Swiss-derived *M. musculus *strains. DNAPars program from Phylip 3.66 was used to generate a parsimony tree from 40.1 kb of sequence data from mouse chromosome 12 derived from six of the seven mouse strains. Because no sequence differences existed between FVB/NT and NIH/Ola these mice were grouped on the tree. The four *M. spretus *strains cluster together. The STF/PAS mice show the greatest divergence within *M. spretus*.

Because this was, to our knowledge, the most sequence data generated from multiple *M. spretus *strains, we were interested in using this information to determine the degree of relatedness between *M. spretus *strains. We therefore conducted maximum parsimony analyses on exon sequence for Spret/EiJ, SEG/PAS, STF/PAS and outbred *spretus *to FVB/NJ, and NIH/Ola utilizing Phylip 3.66 [30]. We did not include FVB/NT in the analysis as we did not identify any sequence differences between FVB/NT and NIH/Ola. We included 40,122 bp from coding and flanking exonic regions across *Skts5 *on mouse chromosome 12 which represented sequence from 90 exons from 35 genes with the identified amino acid alterations and the non-coding polymorphisms within them. DNA variants were all weighted equally. A maximum parsimony tree was generated from the data which was identical to a consensus tree from 100 bootstrap reiterations (Figure 3). As expected, the greatest phylogenetic distance occurred between the *M. musculus *and *M. spretus *groups. The fewest number of polymorphisms occurred between the *M. musculus *strains FVB/NJ and NIH/Ola (FVB/NT) mice. These data suggest that the inbred Swiss-derived strains are more closely related than the wild-derived *M. spretus *strains. Within *M. spretus *strains, Spret/EiJ and outbred *spretus *were the most closely related, and STF/PAS mice showed the most divergence from the other *M*. *spretus *strains. Nonetheless, of the roughly 13,300 amino acids assessed in all four *M. spretus *strains only 0.17% (22 amino acids) were polymorphic between *M. spretus *strains. If *Skts5 *is representative of the genome as a whole, then most single nucleotide polymorphism data available for Spret/EiJ from coding regions will have utility for other *M. spretus *strains.

## Discussion and Conclusion

To our knowledge, this is the largest sequence-based comparison of multiple strains of *M. spretus*. Based on sequence comparison of over 40 kb across a 15-Mb region on chromosome 12, *M. spretus *strains are more closely related to each other than to the Swiss-inbred strains of *M. musculus*. Outbred *spretus *and Spret/EiJ have the highest sequence similarity. This is not unexpected as both of these *M. spretus *mice strains were derived from wild mice isolated from the same region of Spain. STF/PAS mice are derived from mice isolated in Tunisia. This strain showed the highest divergence from the other *M. spretus *strains and was the *M. spretus *strain most closely related to the Swiss-derived *M. musculus *mice. These data fit with the geographical ascertainment of the lines of *M. spretus *as the other *M. spretus *strains all come from Spain.

In this dataset we observed an average of one polymorphism every 130 bp between *M. musculus *and *M. spretus*. This is less than half as frequent as previous reports of sequence variance between *M. musculus *and *M. spretus *[5]. However, this data set was almost entirely restricted to coding regions with only minimal amounts of flanking intronic sequence. Genes are under different mutation constraints than non-functional intragenic regions and therefore show fewer variants [31]. Within the 65 genes analyzed in this study, 31 had no amino acid polymorphisms, 19 genes had one to three amino acid variants and 15 genes had greater than four amino acid variants. Despite the number of non-synonomous changes, the majority of amino acid variants identified in this study were not predicted to show functional consequences using SIFT. Genes and amino acids that showed lower SIFT tolerance scores are being considered as candidates for skin cancer susceptibility.

We conducted this analysis to identify candidate genes for *Skts5*. From our criteria based on the linkage, a number polymorphisms were identified that were consistent with the linkage (Table 4, Additional file 2). These include eight amino acid changes that were found in outbred *spretus *that were not seen in STF/PAS including three that have SIFT scores below 0.10. These variants will be considered as candidates for the linkage. However, the nucleotide driving linkage at *Skts5 *may also be in a non-sequenced regulatory or intragenic region and may affect gene expression or stability. There is also the small possibility that undetected heterogeneity at *Skts5 *exists between the outbred *spretus *mice and we have not sequenced the genome containing the resistance allele. Future studies will address these possibilities.

The five genes that showed the highest number of amino acid polymorphisms (*Cog5*, *Slc26a3*, *Ahr*, *Zfp277*, *1110049B09Rik*) do not share any known function and do not map next to each other at *Skts5*. *Ahr *(aryl hydrocarbon receptor) mediates the toxicity of dioxin compounds, particularly polycylic aromatic hydrocarbons [32]. It has a large number of reported amino acid variants in both mouse and humans. Some variants within *Ahr *have been associated with differences in chemical toxicity in mouse and man [33-35] and in modest effects on cancer risk in humans [36,37]. One of the amino acids identified in our study shows a very low SIFT score and may therefore affect Ahr function, however this variant was observed in all *M. spretus *strains and is therefore not considered to be a candidate for *Skts5*. Two *Ahr *variants, Asn533Ser and Asn544Asp, fit with the linkage data but are not predicted to be functionally significant by SIFT. *Cog5 *is part of the conserved oligomeric Golgi (cog) complex and has been postulated to mediate Golgi trafficking. Two amino acid polymorphisms have been reported in human but none in mouse. Little is known about *Zfp27*. *Slc26a3 *is a transmembrane glycoprotein that transports sulfate in the lower gut. *Slc26a3 *has two amino acids reported in human and none in mouse. The function of *1110049B09Rik *is not known, but the purported human homologue, *FLJ23834*, has a large number of reported amino acid alterations as well (seven in mouse; six in humans). It is possible that the higher rate of amino acid substitution in these genes reflects some underlying selection pressures between *M. musculus *and *M. spretus*.

A number of potentially functional interesting variants were identified that do not fit with the linkage data. The only species in which the *Sostdc1 *amino acid variant Cys15Ser was observed was in *M. spretus*. It is not known if substitution of a cysteine at position 15 with serine in the *M. spretus *isoform results in altered disulfide bond formation, disruption of other secondary structure or other functional consequence. Another interesting polymorphism is in *4921508M14Rik*. This gene contains a stop codon at position 109 in all of the *M. spretus *strains theoretically resulting in a truncated protein missing the 37 terminal amino acids. The function of this gene is unknown. *EG629820*, which contains several amino acid variants thought to be functional by SIFT, is a predicted gene that has shown expression differences of unknown biological consequence in a number of murine studies [38].

There was a relatively low degree of sequence variation between the *M. spretus *strains; however we identified twenty-two changes that resulted in intra-*spretus *amino acid polymorphisms. Thus, based on this dataset, nearly twenty percent of the amino acid alterations identified between *M. spretus *and *M. musculus *are polymorphic within *M. spretus*. In contrast, sequencing results from the two Swiss-derived *M. musculus *strains identified only one amino acid that was polymorphic between the FVB/NJ strain and NIH/Ola. Sequence comparison of three closely related *M. musculus s*trains: NIH/Ola, FVB/NT and FVB/NJ revealed only six of 140,448 basepairs (0.004%) that were polymorphic between NIH/Ola and FVB/NJ. There were no sequence differences identified between FVB/NT and NIH/Ola. Because sequence variants exist between FVB/NT and FVB/NJ, researchers comparing results across studies should be aware of which strain of FVB/N is used. Our results confirm previous studies in which variations in coding regions were more common in wild-mice than in laboratory-derived strains [7], perhaps reflecting differences in breeding and selection pressure.

Our data comparing sequence diversity between *M. spretus *strains may not be representative of genome as a whole since this study was limited to one locus on chromosome 12 and included only genes, predicted coding elements, and small amounts of flanking intronic sequences. In this study, we conducted our sequence comparisons by analyzing only exons in which we initially identified an amino acid variation between outbred *spretus*, Spret/EiJ, FVB/N, and NIH/Ola. There may be additional amino acid variations at *Skts5 *in which Spret/Ei*J*, outbred *spretus *and NIH/Ola share an allele that is different from the other strains of *M. spretus*. We also cannot rule out the possibility that *Skts5 *may have unique genomic properties compared to other loci. Previous studies have reported different phylogenetic trees when comparing sequence samples from across the genome (global comparison) with gene-specific phylogenetic trees (local comparison)[39]. *Skts5 *may represent a region with unique local characteristics between *M. spretus *strains. For instance, *Skts5 *may be under differential evolutionary constraints against sequence divergence in *M. spretus *strains. Nonetheless, our data suggest that genomic data and single nucleotide markers in the database for Spret/EiJ, particularly intragenic SNPs, are more likely to be shared with other *M. spretus *strains than with *M. musculus *strains, increasing the utility of the publicly available SNP maps for studies utilizing any *M. spretus *mouse.

## Methods

### Animal material

The ancestral and laboratory origins of each of the mice in the study are as follows: SEG/PAS (Grenada, Spain, inbred line maintained by the Institute Pasteur), STF/PAS (Fondouk el Djedid, Tunisia, inbred line maintained by the Institute Pasteur), Spret/EiJ (Puerto Real, Ibiza, Spain, inbred line maintained by the Jackson Laboratories), outbred *spretus *(Spain, outbred line maintained by Stephen Brown, PhD of the Medical Research Council, Harwell, England), FVB/NT (Switzerland, inbred line maintained by Taconics, US), FVB/NJ (Switzerland, inbred line maintained by the Jackson laboratories) and NIH/Ola (Switzerland, inbred line maintained by Harlan Olac, UK). DNA was isolated from tails or spleens of outbred *spretus*, NIH/Ola and FVB/NT mice using standard methods [40]. Spret/EiJ and FVB/NJ DNA was purchased from the Jackson Laboratories. STF/PAS and SEG/PAS DNAs were a gift from Xavier Montagutelli, DVM, PhD of the Institut Pasteur.

### Linkage analysis and genotyping of markers at Skts5

To refine linkage at *Skts5 *in the outbred *spretus *× NIH/Ola backcross ten additional single nucleotide polymorphisms from chromosome 12 were genotyped in mice at Applied Biosystems. Genotypes of the new markers were combined with previously genotyped microsatellite markers D12Mit182, D12Mit242, D12Mit60, D12Mit153, D12Mit154, D12Mit2, D12Mit68, and D12Mit5. Quantitative trait locus analysis using WinQTLCart [41] was conducted on the genotypes of the 18 markers on 351 intercross mice using skin papilloma number as the trait. To rule out genetic heterogeneity within outbred spretus mice at *Skts5 *(15–19 cM), we genotyped seven additional markers (D12Mit243, D12Mit187, D12Mit146, D12Mit186, D12Mit222, D12Mit61 and D12Mit62) in six different outbred *spretus *fathers, Spret/EiJ, NIH/Ola, and NIH/Ola × outbred *spretus *F1 and STF/PAS.

### PCR primer design, sequencing and analysis

Intron/exon boundaries of all genes and coding regions mapping to *Skts5 *were identified using the Ensembl database, builds 35–48. When multiple splice forms of a gene were present, exons from all splice forms were included in the analyses. We designed PCR primers using Integrated DNA Technology's SciTools PrimerQuest web-based program [42]. Primer sequences and PCR conditions are listed in Additional file 4. PCR products were treated with Exo/SAP-IT to remove single stranded DNA (USB). Automated sequencing of PCR products was conducted on an ABI 3700 by standard methods. Primers used for PCR were also used for the sequencing. Forward and reverse sequences were analyzed and compared using DNAstar 3.0. The sequence traces were inspected visually whenever a nucleotide substitution was indicated. When a polymorphism was identified between FVB/NJ and Spret/EiJ, PCR products from additional strains NIH/Ola, FVB/NT, outbred *spretus*, SEG/PAS and STF/PAS were sequenced.

### Evolutionary comparison

Sequence alignments between *M. musculus *and other species were obtained from the Ensembl database when homologies were present. Tolerance scores were generated using the SIFT program and entering in the NCBI protein G numbers for each *M. musculus *gene at *Skts5 *[29]. SIFT sorts intolerant amino acid substitutions from tolerant substitutions by protein based alignments from other species. A tolerance score of 0 to 1.0 is generated for each amino acid variant. Low tolerance indices (less than 0.05) are suggested of non-tolerated (deleterious or functional) amino acid substitutions. Higher tolerance indices indicate that an amino acid substitution is less likely to have functional consequences.

### Splicing Studies

RNA was isolated from tails of NIH/Ola, Spret/EiJ and outbred *spretus *mice using standard Trizol methods per manufacturer's recommendations (Invitrogen). One microgram of RNA was reversed transcribed using the Iscript cDNA synthesis kit (Biorad) according to manufacturer's directions. PCR primers were designed in exons flanking the potentially aberrantly spliced exon. Primer sequences and PCR conditions are listed in Additional file 4. Genomic DNA from the mice was used as a negative control. PCR products were run on 1.5% agarose gels for comparison of product sizes.

### Phylogenetic comparison

Maximum DNA parsimony analyses were conducted using sequence from exons that were sequenced in all strains of mice using the DNAPars program included in the Phylogeny Interference Package (Phylip) version 3.66 [30]. All nucleotide changes were weighted equally. To assess support for the most parsimonious tree, we also performed a bootstrap analysis and compared the resulting consensus tree to the most parsimonious tree. This resulted in an identical tree. We used the program Drawgram in Phylip 3.66 to construct a phylogenetic tree from this data without using branch length considerations.

## Abbreviations

Phylip: Phylogeny Interference Package; QTL: Quantitative trait loci; *Skts5*: *Skin tumor susceptibility 5*; SIFT: Sorting tolerant from intolerant.

## Authors' contributions

KLM participated in sequence analysis, phylogenetic analysis and organization and interpretation of data. JLF, HYC, NG and AMD contributed to sequence analysis and organization of data. AB and JHM contributed to linkage data. AET conceived the study, participated in data analysis and coordination and helped to draft the manuscript. All authors participating in editing of the final manuscript. All authors read and approved the final manuscript.

## Supplementary Material

Additional file 1 **Table 1: Genes mapping to Skts5.** This table shows a list of all genes mapping to Skts5 including their Ensembl reference number. Click here for file

Additional file 2 Table 2: All sequence variants identified at *Skts5*. This table shows all the sequence variants identified in the study, the strain of mouse in which it was found, the amino acid change if applicable, and the rs number if previously entered in dbSNP. Click here for file

Additional file 3 **Table 3: Genes showing strong conservation across species.** This table shows a list of amino acid changes that appear to be strongly conserved across species. In red are the amino acids that match M. musculus and in blue are the amino acids that match M. Spretus. The corresponding amino acids in nine species as well as the mouse strains sequenced in the study are shown. Click here for file

Additional file 4 **Table 4: Primers for each exon for sequencing and splice site analysis.** This table lists all forward and reverse primers used for sequencing in this study. Click here for file
